# Transcriptomic regulation of seasonal coat color change in hares

**DOI:** 10.1002/ece3.5956

**Published:** 2020-01-15

**Authors:** Mafalda S. Ferreira, Paulo C. Alves, Colin M. Callahan, Iwona Giska, Liliana Farelo, Hannes Jenny, L. Scott Mills, Klaus Hackländer, Jeffrey M. Good, José Melo‐Ferreira

**Affiliations:** ^1^ CIBIO Centro de Investigação em Biodiversidade e Recursos Genéticos InBIO Laboratório Associado Universidade do Porto Vairão Portugal; ^2^ Departamento de Biologia Faculdade de Ciências da Universidade do Porto Porto Portugal; ^3^ Wildlife Biology Program University of Montana Missoula MT USA; ^4^ Division of Biological Sciences University of Montana Missoula MT USA; ^5^ Amt für Jagd und Fischerei Graubünden Chur Switzerland; ^6^ Office of Research and Creative Scholarship University of Montana Missoula MT USA; ^7^ Institute of Wildlife Biology and Game Management BOKU—University of Natural Resources and Life Sciences Vienna Austria

**Keywords:** developmental timeline, gene expression, molt cycle, seasonal coat color change, transcriptomics

## Abstract

Color molts from summer brown to winter white coats have evolved in several species to maintain camouflage year‐round in environments with seasonal snow. Despite the eco‐evolutionary relevance of this key phenological adaptation, its molecular regulation has only recently begun to be addressed. Here, we analyze skin transcription changes during the autumn molt of the mountain hare (*Lepus timidus*) and integrate the results with an established model of gene regulation across the spring molt of the closely related snowshoe hare (*L. americanus*). We quantified differences in gene expression among three stages of molt progression—“brown” (early molt), “intermediate,” and “white” (late molt). We found 632 differentially expressed genes, with a major pulse of expression early in the molt, followed by a milder one in late molt. The functional makeup of differentially expressed genes anchored the sampled molt stages to the developmental timeline of the hair growth cycle, associating anagen to early molt and the transition to catagen to late molt. The progression of color change was characterized by differential expression of genes involved in pigmentation, circadian, and behavioral regulation. We found significant overlap between differentially expressed genes across the seasonal molts of mountain and snowshoe hares, particularly at molt onset, suggesting conservatism of gene regulation across species and seasons. However, some discrepancies suggest seasonal differences in melanocyte differentiation and the integration of nutritional cues. Our established regulatory model of seasonal coat color molt provides an important mechanistic context to study the functional architecture and evolution of this crucial seasonal adaptation.

## Graphical abstract

We analyze skin transcription changes during the autumn color molt of the mountain hare and integrate the results with an established model of gene regulation across the spring molt of the closely related snowshoe hare. Our work provides an important mechanistic context to study the functional architecture and evolution of this crucial seasonal adaptation.

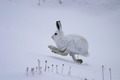

## 1 INTRODUCTION

Seasonal environments impose numerous challenges to organismal survival. To track seasonality, individuals cycle important biological processes, known as phenologies (e.g., reproduction, migration, hibernation, or molt), allowing a better phenotypic match with seasonal selective pressures (Helm et al., 2013; Visser, Caro, Oers, Schaper, & Helm, 2010). Accurate timing of physiological and behavioral changes is important for fitness. Synchronizing phenotypes with the environment requires a precise perception of predictable cues (e.g., variation in day length) that anticipate seasonal change and trigger regulatory cascades and shifts in gene expression, with consequent phenotypic change (Bradshaw & Holzapfel, 2007). Quantifying gene expression changes underlying phenological traits is therefore crucial to understand how organisms synchronize their phenotypes with the surrounding environment (Ferreira et al., 2017; Giska et al., 2019; Jones et al., 2018; Schwartz & Andrews, 2014).

Discrete seasonal molts have evolved as energy‐efficient phenological adaptations to regenerate hair layers and produce coats with insulating properties adequate for each season (Geyfman, Plikus, Treffeisen, Andersen, & Paus, 2015; Hart, 1956; Ling, 1972). In at least 20 vertebrate species, seasonal molts are accompanied by the change from summer brown to winter white coats, allowing camouflage in snow‐covered environments (Mills et al., 2013, 2018). As in other phenological traits, seasonal molting is mostly triggered by annual changes in day length (photoperiod) (Bissonnette & Bailey, 1944; Hoffmann, 1978; Lincoln, Clarke, Hut, & Hazlerigg, 2006; Zimova et al., 2018). Other environmental factors, such as temperature or snow presence, have been suggested to influence the rate and completeness of the molt in different mammals (Zimova et al., 2018 and references therein). However, molt onset seems to have minimal plasticity (Zimova, Mills, Lukacs, & Mitchell, 2014), suggesting that photoperiod is the most important cue entraining annual molt timing. Consequently, since animals coordinate molt with the fixed variation in photoperiod, the reductions of annual snow cover associated with climate change are predicted to lead to increased mismatches between coat and background colors, disrupting camouflage and negatively affecting the fitness of seasonally color changing species (Atmeh, Andruszkiewicz, & Zub, 2018; Zimova, Mills, & Nowak, 2016). Predicting how color changing molts will respond to environmental change requires a better understanding of the internal mechanisms that regulate the change of hair and color (Helm & Stevenson, 2014).

In mammals, pathways and genes involved in the perception of the photoperiodic signal, such as the circadian clock and melatonin production and reception, hair growth cycle, and melanogenesis, are expected to be involved in the regulation of seasonal coat color changing molts (Allain & Rougeot, 1980; Balsalobre, 2002; Duncan & Goldman, 1984; Ferreira et al., 2017; Lin et al., 2009; Lincoln et al., 2006; Slominski, Tobin, Shibahara, & Wortsman, 2004). The molecular mechanisms underlying the hair growth cycle (Lin et al., 2009; Schlake, Beibel, Weger, & Boehm, 2004; Slominski et al., 2004) and the basis of static melanin‐based coloration have been thoroughly studied (Allain & Rougeot, 1980; Duncan & Goldman, 1984; Logan & Weatherhead, 1980). However, the specific regulatory mechanisms of coat color changing molts have only been addressed recently. We previously studied the regulatory patterns of seasonal changing molts in snowshoe hares, inferring gene expression changes in the skin during the spring white‐to‐brown molt in the species using RNA‐sequencing (Ferreira et al., 2017). This work established a genic regulatory model for the seasonal molt of snowshoe hares characterized primarily by genes involved in the hair growth cycle, circadian rhythms, and pigmentation. For example, among the differentially expressed genes along the molt, a pulse of expression of the agouti signaling protein (*ASIP*) gene was detected at the onset of the molt. The ASIP peptide signals the switch between darker and lighter melanin pigment, suggesting a role of the melanin production pathway in the regulation of seasonal color change (Ferreira et al., 2017). Furthermore, *cis*‐regulatory variation at *ASIP* has been linked to polymorphism in winter coat color in snowshoe hares (*Lepus americanus*) (Jones et al., 2018) and mountain hares (*L. timidus*) (Giska et al., 2019). It remains unclear how generalizable these results are across species.

Here, we study the transcriptional landscape of the autumn molt in the mountain hare (*L. timidus*) and integrate the results with the previously quantified gene expression landscape of the spring molt of snowshoe hares (Ferreira et al., 2017). Mountain hares are widely distributed across Eurasia, from Western Europe to Eastern Russia, with some isolated populations in the Alps, Ireland, British, and Faroe Islands (Angerbjörn, 2018). Throughout most of their range, mountain hares alternate between summer brown and winter white coats, with some exception, such as in Ireland and the Faroe Islands (Angerbjörn, 2018; Bergengren, 1969; Mills et al., 2018; Schai‐Braun & Hackländer, 2016). Here, we test the generality of the snowshoe hare seasonal molt regulatory model (Ferreira et al., 2017), placing hair color change in the stages of the hair growth cycle, and pin‐pointing seasonal differences in regulatory mechanisms between the two species. While snowshoe and mountain hares are not sister species (Matthee, Vuuren, Bell, & Robinson, 2004; Melo‐Ferreira et al., 2012), it is yet unclear to what degree ancestral variation or ancient introgression may explain sharing of the trait (Jones et al., 2018). We predict that the major expression waves related with pelage molt are conserved in these two species given the conservation of hair growth cycle mechanism in mammals (Lin, Chudova, Hatfield, Smyth, & Andersen, 2004; Schlake et al., 2004; Stenn & Paus, 2001). Possible differences may then be related to species‐specific differences or seasonal differences in hair color and composition, or to the integration of seasonal specific cues onto the regulation of hair growth. By integrating results across both species, we expand the model of genic regulation during both autumn and spring seasonal coat color change in hares and provide a context to understand the functional mechanisms controlling this crucial seasonal adaptation.

## 2 MATERIALS AND METHODS

### 2.1 Sampling, laboratory procedures, and sequencing

**Figure 1:**
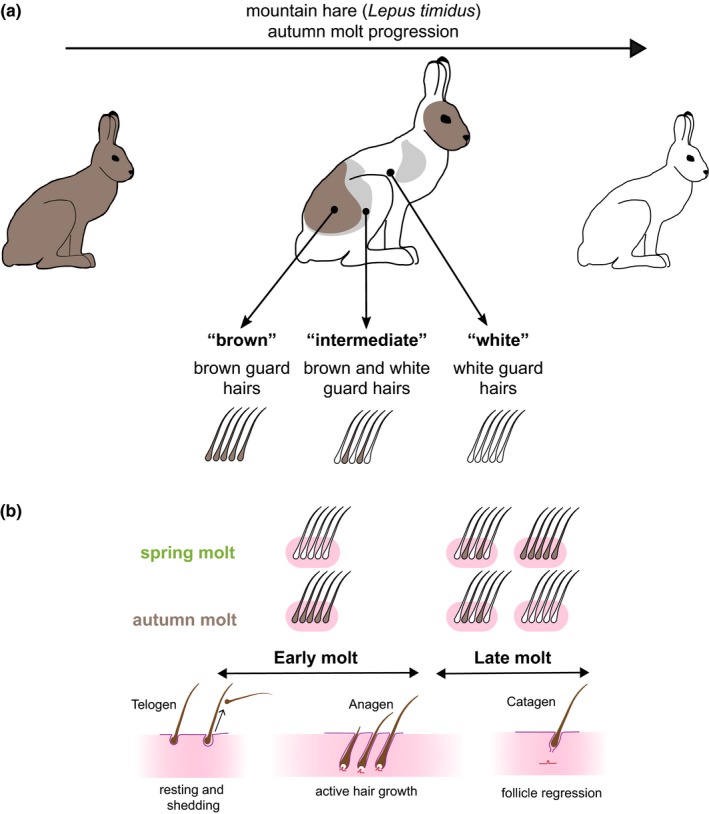
Mountain hare autumn molt and sampling scheme. (a) Three patches of skin were sampled from individuals undergoing autumn molt, classified according to the color of guard hairs: “brown,” “intermediate” (both brown and white hairs), and “white.” (b) Parallel between the molt stages of our sampling scheme and the stages of the hair growth cycle, as established for the spring molt in Ferreira et al., (2017), and hypothesized for the autumn molt (this work)

Sampling was performed following the reasoning of Ferreira et al., (2017). Briefly, the spatial progression of the molt across the body of a single molting individual results in the simultaneous occurrence of different skin patches that are expected to be in different molting stages—proliferative stage of active hair growth “anagen,” the follicle regression stage “catagen” and a final resting stage “telogen” (Geyfman, Gordon, Paus, & Andersen, 2012; Lin et al., 2004; Schlake et al., 2004) (Figure 1). Given an expected lag between expression changes and the visible changes in hair color used as proxy of molting stage (Ferreira et al., 2017), we expect that early molt (“brown”) captures mostly anagen‐related processes and possibly late telogen, and late molt (“white”) captures late anagen and the transition to catagen (Figure 1b). Four mountain hare (*L. timidus*) specimens were collected from hunting bags during regular permit hunting in Grisons, Switzerland (Table S1). Hares were undergoing pelage molt, from brown to white, which starts on the extremities and ends in the head and dorsum, a pattern that is reversed during spring molt (Hewson, 1958). Sampling different molting stages in the same individual allows controlling for individual variation in gene expression (Ferreira et al., 2017), and only individuals with three identifiable patches of skin corresponding to the stages of molt progression were used in this study: early molt (brown hair), intermediate stage (both brown and white hair), and late molt (white hair) (Figure 1), resulting in a total of 12 tissue samples. Skin patches of 2 cm^2^ were collected from the animals’ dorsum for each skin type immediately after death, and stored first in RNAlater and then at −80°C until RNA extraction.

From each skin sample, total RNA was extracted using the RNeasy® Mini Kit (QIAGEN) and strand‐specific cDNA libraries were synthetized using the SureSelect Strand‐Specific RNA Library Prep for Illumina Multiplexed Sequencing (Agilent Technologies). Library sizes were inferred using Bioanalyzer 2100 and pooled in equal molar concentrations using the KAPA Library quantification kit (KAPA BIOSYSTEMS). Pooled libraries were sequenced in two separate Illumina HiSeq runs: ~3% of two lanes of 100 bp paired‐end reads of an HiSeq 2000, at University of California Berkeley, and then three lanes of 101 bp paired‐end reads in a HiSeq 2500 at Utah Microarray Core.

### 2.2 Quality control and transcriptome assembly

Reads failing quality control, flagged by the Illumina Casava‐1.8 Fastq Filter (Gordon, 2011), were discarded and adapters removed using Cutadapt (1.3). Trimmomatic (0.32) was used to retain a minimum phred quality score of 15 across 4 bp sliding windows, to trim the first 13 base pairs from every read, which showed a nucleotide composition bias (Hansen, Brenner, & Dudoit, 2010), and finally to remove trimmed reads smaller than 23 bp.

A de novo skin transcriptome assembly for *L. timidus* was performed to allow an independent unbiased comparison with the results of Ferreira et al., (2017). The transcriptome assembly was performed with Trinity (2.6.6) (Grabherr et al., 2011), using filtered and properly paired reads and specifying “RF” for strand‐specific assembly. Transrate (1.0.2) (Smith‐Unna, Boursnell, Patro, Hibberd, & Kelly, 2016) was used to assess the quality of the transcriptome, filtering erroneously assembled contigs. We then annotated this filtered transcriptome using the rabbit (OryCun2.0) and mouse (GRCm38.p6) ENSEMBL 92 peptide references applying the reciprocal blast implemented in Transrate. The mouse annotation was only used when no rabbit annotation was produced for all analyses, except for the Reactome pathway enrichment analysis (see below).

### 2.3 Differential expression analysis

Reads were mapped to the de novo assembled transcriptome using bowtie2 (2.3.4.1) with the following flags: ‐q, ‐‐phred33, ‐D 20, ‐R 3, ‐N 1, ‐L 20, ‐i S,1,0.50, ‐‐dpad 0, ‐‐gbar 9,999,999, ‐‐mp 1,1, ‐‐np 1, ‐‐score‐min L,0,‐0.1, ‐I 1, ‐X 1,000, ‐‐no‐mixed, ‐‐no‐discordant, ‐‐nofw, ‐p 1, ‐k 200, ‐‐fr, ‐x. To generate bowtie2 indices, we used the *extract‐transcript‐to‐gene‐map‐from‐trinity* script and then *rsem‐prepare‐reference* script from RSEM (1.3.0). Finally, the outputs from bowtie2 were used as inputs for *rsem‐calculate‐expression* to generate relative abundances for all genes across the transcriptome. The transcriptome annotation was used to filter the RSEM output, removing Trinity “genes” (putative genes) with annotation to multiple genes (i.e., assembled putative isoforms annotated to different ENSEMBL genes), or with no annotation.

Using the filtered RSEM output, differential expression was inferred between molt stages (“brown,” “intermediate,” and “white”) using *edgeR* (3.20.9) (Robinson, McCarthy, & Smyth, 2010). Only Trinity genes expressed at a minimum of one count per million (CPM) mapped reads in at least 4 samples were retained. The trimmed mean of M‐values was used to normalize data across libraries, and a Cox‐Reid profile‐adjusted likelihood was used to calculate common, trended, and tagwise dispersions, as described in Robinson et al. (2010). The biological coefficient of variation (BCV) was estimated as the square root of the common dispersion (McCarthy, Chen, & Smyth, 2012). To inspect the relationship between expression counts in each sample, we plotted a multidimensional scaling plot (MDS plot) in R using the 500 genes with highest dispersion between each sample pair. Given an aberrant expression pattern estimated for one individual, which did not result in the individualization of molting stages (Figure S1) expected from our sampling strategy (Ferreira et al., 2017), we evaluated the BCV and estimated the expected power to detect gene expression changes in the 4 versus 3‐individual dataset using *RNASeqPower* (Hart, Therneau, Zhang, Poland, & Kocher, 2013). For comparison, we also estimated the expected power for the snowshoe hare dataset from Ferreira et al., (2017).

The inspection of the MDS plot showed that replicates tend to cluster primarily by molting stage and secondarily by individual (Figure S2), suggesting that individual is a significant contributor of variation to gene expression differences. To incorporate this factor in the analysis, we used “individual” as a blocking factor in the statistical test for differential expression, to control for individual variation in gene expression. Differential expression between molting stages was inferred using a likelihood ratio test for 3 pairwise comparisons. After a Benjamin‐Hochberg multiple test correction, all genes with a false discovery rate (FDR) smaller than 0.05 were considered differentially expressed. We tested for significant overlap between mountain hare and snowshoe hare set of upregulated genes in each molt stage, by collapsing the upregulated genes for the same stage from each pairwise comparison, and using a Fisher exact test in R, considering as background all common unique ENSEMBL gene annotations between the transcriptomes of the two species.

A clustering analysis was then performed to identify major expression patterns across genes. We converted CPM levels for each gene to fragments per kilobase per million reads mapped (FPKM) and further log2 transformed and mean centered these values. The log2 mean‐centered FPKM value for each differentially expressed gene was used as input for two clustering methods. First, a complete linkage hierarchical clustering was performed using the Pearson's correlation coefficient between samples and Euclidian distances between genes with the R package *gplots* (3.0). Second, a partition clustering analysis was done using a gap statistic to calculate the optimal number of clusters (k) using *clustgap( )* from the R package *cluster* (2.0.7). The optimal k of 9 was used in a partitioning around medoids (pam) clustering analysis using *pam( )* from the R package cluster (Figure S3). The k clusters were then summarized in 3 major general patterns of expression, and the pam clustering was repeated with k = 3. Finally, the expression levels and mean expression level per cluster were plotted using a modified version of the *plot_expression_patterns.pl* script from the Trinity pipeline.

### 2.4 Functional enrichment analysis

The functions of genes within each cluster of distinct expression signature were investigated using a Gene Ontology enrichment analysis using Ontologizer (2.1) (Bauer, Grossmann, Vingron, & Robinson, 2008). A custom gene association file (GAF) was built using custom scripts *make_input_4_gaf.py* and *do_Gaf_file_new_version.py* (available at https://github.com/evochange/coat_color_change_transcriptomics) and using the GO term ID for each ENSEMBL ID present in the transcriptome assembly. Enrichment was tested (FDR < 0.05) using a parent‐child‐union test with the entire skin transcriptome as background and the Benjamin–Hochberg correction for multiple tests. Furthermore, a Reactome pathway enrichment analysis was done using the Reactome database (version 65; https://reactome.org/PathwayBrowser/#TOOL=AT) and the mouse annotation for each gene (the rabbit is not supported in the database). Enrichment was tested using the binomial test.

Finally, we investigated the expression profiles of genes annotated with Gene Ontology terms potentially related with seasonal coat color change: “molting cycle” (GO:0042303), “circadian rhythm” (GO:0007623), “pigmentation” (GO:0043473), or one of the child terms. The child terms were identified using the GOOSE tool at the Gene Ontology Consortium website (http://amigo.geneontology.org/goose).

### 2.5 Quantitative PCR for Agouti gene expression

Given its importance as a marker of anagen and for coat color regulation (Ferreira et al., 2017; Giska et al., 2019; Jones et al., 2018), we further investigated *ASIP*’s expression pattern. We quantified the expression of *ASIP* hair cycle isoform and reference housekeeping genes *SDHA* and *ACTB* for the three individuals and nine skin samples included in the differential gene expression analyses. Total RNA was extracted from 30 mg of each skin sample with the RNeasy® Mini Kit (QIAGEN). Tissue was homogenized in a rotor‐stator homogenizer (Mixer Mill MM400, Retsch) at 30 Hz for 10 min. The RNA integrity was checked with the Agilent Technologies TapeStation for a minimum RIN > 7. First‐strand cDNA synthesis was performed using oligo (dT) primers and the GRS cDNA synthesis kit (GRISP) using 400 ng of RNA from each skin sample. Relative expression was quantified based on quantitative PCR (qPCR) of the gene of interest normalized across individuals to the reference genes (primers are indicated in Table S2). Their amplification efficiencies were calculated based on the slope of a regression line fitted to C_t_ values of five samples from 2‐fold serial dilutions (E = 10^(−1/slope)^ − 1), using two replicates per sample. Then, to quantify *ASIP* expression across the molt, three replicates of qPCR reactions per skin sample were performed using 1X iTaq^TM^ universal SYBR^®^ green supermix (Bio‐Rad), 0.4 µmol/L each primer and 1 µl cDNA (2‐fold dilution of stock cDNA), total volume 10 µl, and the thermal conditions shown in Table S3. The raw qPCR results, presented as the threshold cycle (C_t_), were used to calculate expression of the *ASIP* hair cycle isoform, normalized by the expression of the *ACTB* or *SDHA* gene according to the formula 2 ${}^{-\Delta C_{t}}$, where $\Delta C_{t}=C_{t}^{\mathrm{Agouti}}-C_{t}^{\text{ref}\;\text{gene}}$. We tested for differences in expression between molt stages using the linear regression (*pcr_lm()*) test from R package *pcr* (Ahmed & Kim, 2018), using “individual” and “molt stage” as factors, and *ACTB* as the reference gene.

## 3 RESULTS

### 3.1 Assembly, annotation, and differential expression

A total of 139,005,571 paired‐end reads were obtained, averaging 11,583,798 read pairs per individual (range = 10,974,557–12,255,152). After adapter and quality‐based trimming, the final dataset comprised 124,393,164 read pairs (averaging = 10,366,097 read pairs per individual, range = 9,916,123–10,946,710), varying in length from 23 to 87 base pairs (Table S4). These reads were then used to produce a strand‐specific assembly using Trinity, and the resulting raw transcriptome consisted of 173,848 Trinity “genes” and 233,182 Trinity “transcripts” (Table S5). After quality control using Transrate, the transcriptome contained 159,769 “genes” and 202,135 “transcripts” (Tables S5 and S6). Finally, the annotation‐based filter resulted in 36,101 Trinity “genes” (Table S6).

The inspection of the MDS plot revealed that the skin samples of one individual consistently deviated from the remaining samples and did not capture the individualization of the sampled molt stages aimed with our sampling strategy (Figure S1) (Ferreira et al., 2017). This suggests an artificial bias during sampling that increased the variance in the dataset. The 3‐individuals’ dataset provided higher power (0.82 vs. 0.72 to detect a 1.5‐fold change for a probability of false positives of 0.05) and lower BCV (0.16 vs. 0.21) when compared to the 4‐individuals’ dataset. This estimated power is comparable to that of the snowshoe hare dataset from Ferreira et al., (2017) (0.85 to detect 1.5‐fold changes in expression), and adequate to detect differential expression for lowly expressed genes (Todd, Black, & Gemmell, 2016). Therefore, hereafter we report the results from the analyses of the 3‐individuals’ dataset. After filtering for multiple annotated and lowly expressed genes, our transcriptome consisted of 17,270 genes, corresponding to 12,070 annotated ENSEMBL genes (11,223 rabbit genes and 847 mouse genes) (Tables S6 and S7). Of the annotated genes, 75% (9,091) were also present among the 10,345 genes annotated in the snowshoe hare transcriptome (Ferreira et al., 2017).

**Figure 2:**
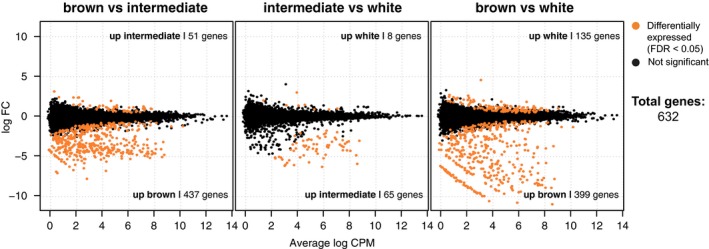
Smear plots showing the number of differentially expressed genes (FDR < 0.05) in three pairwise comparisons between molt stages. Average log counts per million for each gene (dots) are plotted against log fold change. For each comparison, the number of upregulated genes in each stage is shown

**Figure 3:**
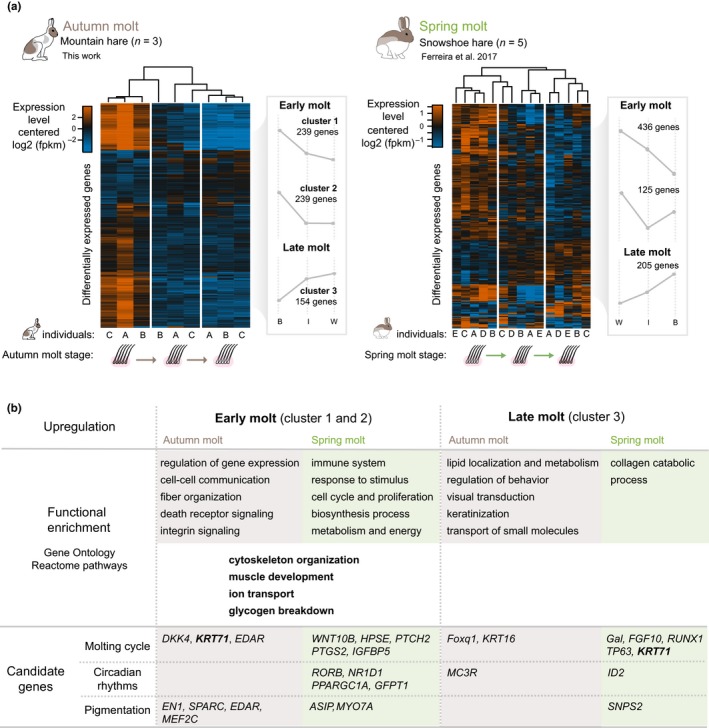
A model of the transcriptional landscape of seasonal coat color change. Left panels show the results of this work on the autumn molt of the mountain hare and right panels show the corresponding results on the spring molt of the snowshoe hare, adapted from Ferreira et. al (2017). (a) Patterns of expression of differentially expressed genes in the mountain hare and snowshoe hare datasets are shown as heatmaps of log2 transformed, mean‐centered FPKM levels of expression per differentially expressed gene (row) and sample (column; a–c and a–e). Dendrograms represent the complete linkage hierarchical clustering of Pearson's correlation coefficient between samples. Clusters result from PAM clustering of individual gene expression levels for k = 3 (B—“brown”; I—“intermediate”; W—“white”). The gray line connects the mean expression value of genes’ expression in “brown” (B), “intermediate” (I), and “white” (W). (b) Functional enrichment and candidate gene expression at early and late molt. For clusters in (a), a Gene Ontology and Reactome enrichment analysis was performed, and the presence of candidate genes, known to have functions related with molting cycle, circadian rhythms, and pigmentation regulation was inspected. Terms and genes specific to mountain hare (this work) and snowshoe hare (Ferreira et al., 2017) are highlighted in brown and green, respectively. Bold black indicates common terms to both species

Differential expression was tested in three pairwise comparisons between the “brown,” “intermediate,” and “white” molt stages, and 632 genes, corresponding to 514 unique ENSEMBL annotations, were found differentially expressed in at least one pairwise test (Figure 2; Table S8). Of these, 131 (25%) were also found differentially expressed in the snowshoe hare dataset (total of 754 unique ENSEMBL annotations) in any stage, representing a significant overlap (Fisher exact test, odds ratio = 4.37, *p* = 2.2E‐16; Table S9). A significant overlap was also found among genes upregulated early in molt in both mountain and snowshoe hares (102 unique ENSEMBL annotations of 379 and 547 upregulated early in mountain and snowshoe hare, respectively; Fisher exact test, odds ratio = 6.83, *p* = 2.20E‐16), but no overlap or significant overlap among genes upregulated in intermediate or late in molt, respectively (*p* > .05; Table S9). As in Ferreira et al., (2017), most upregulated genes were found in the early molt (“brown” in the autumn molt), whereas “intermediate” was the stage with the lowest number of upregulated genes (Figures 2, 3a, and S4).

To inspect the pattern of expression of these genes across the molt, we plotted mean‐centered log2 FPKM expression levels in a heatmap (left panel of Figure 3a), showing that most genes tend to be upregulated early in the molt and that levels of expression tend to decrease toward the end of molt. However, a smaller proportion of genes showed higher levels of expression at the end of molt. The hierarchical clustering analyses showed that sampled “brown” and “white” skin tissues clustered according to molt stage, while “intermediate” samples were split between the two clusters. Furthermore, the partitioning clustering analysis resulted in three clusters of genes with similar expression patterns: 1) 239 genes that decrease their expression gradually toward the end of molt, from “brown” to “white”; 2) 239 genes decrease their expression from “brown” to “intermediate” and retain a constant level of expression toward “white”; and 3) 154 genes increase their expression gradually from “brown” to “white” (left panel of Figure 3a and S4). Both clusters 1 and 2 group genes with higher expression early in the molt, which was also found in snowshoe hares (right panel of Figure 3a).

### 3.2 Gene Ontology term and Reactome pathway analyses

To understand if genes with different expression patterns could be distinguished by their functions, we tested the enrichment of Gene Ontology (GO) terms and Reactome pathways in each gene cluster obtained from partitioning clustering analysis (Tables S10–S15). We found 65, 4, and 2 GO biological process terms enriched in cluster 1, 2, and 3, respectively (Figure 3b, Tables S10–S12). Furthermore, 10 Reactome pathways were found enriched in cluster 1, 18 in cluster 2 and 9 in cluster 3 (Figure 3b, Tables S13–S15). In general, the enrichment analysis revealed functions and pathways that are expected in the molting skin (Ferreira et al., 2017). Both clusters with higher levels of expression in “brown” (1 and 2; Figure 3b) showed a similar enrichment of GO terms that were related with muscle development, cytoskeleton organization, transmembrane transport, circulatory system development, cell–cell communication and adhesion and regulation of gene expression and protein synthesis (Figure 3b, Tables S10 and S11). Pathway enrichment in both clusters was consistent with the GO enrichment; muscle‐related pathways enriched in cluster 1, and metabolism of proteins and RNA, and signaling transduction were enriched in cluster 2 (Figure 3b, Tables S13 and S14). In cluster 3 (genes increasing their expression toward the end of molt), only two GO biological process terms were found enriched: “lipid localization” and “regulation of behavior” (Figure 3b, Table S12). Pathways found enriched in cluster 3 are involved in keratinization, lipid metabolism, the retinoid cycle, and transport of small molecules (Figure 3b, Table S15). Next, we compared patterns of functional enrichment detected in mountain hares with patterns found during the snowshoe hare molt (Ferreira et al., 2017). Overlapping GO terms and Reactome pathways were only detected for gene clusters showing higher expression early in the molt cycle including muscle tissue development, ion transport, cytoskeleton organization, and glycogen breakdown (Figure 3b and S5).

In addition, we inspected the expression of genes with functions expected to be involved in seasonal coat color change. We found nine candidate genes that were differentially expressed across the molt with the GO terms “pigmentation,” “molting cycle,” and “circadian rhythm” (12 Trinity genes; Figure 3b, Table S16). Six of these genes (*EN1, SPARC, MEF2C, DKK4, KRT71, EDAR*) showed higher expression at early molt (cluster 2) and were found to be upregulated in “brown,” while three (*Foxq1, KRT16*, *MC3R*) increased their expression toward the end of molt (cluster 3) and were up regulated either in “white” or “intermediate” (Figure 3b, Table S16). Genes *Foxq1, DKK4, KRT71, KRT16, EDAR* are putatively involved in molting cycle‐related functions. Genes *EN1, SPARC, EDAR,* and *MEF2C* are annotated to the Gene Ontology term “pigmentation” and the child term “melanosome differentiation,” being all upregulated in “brown.” Finally, one gene, *MC3R,* annotated to child terms of “circadian rhythm” (“circadian regulation of gene expression” and “locomotor rhythm”), showed higher expression in “white” and “intermediate.” *KTR71* was the only candidate gene found differentially expressed both here and in Ferreira et al., (2017) (an overlap not expected by chance; odds ratio = 66.3, *p* = .02; Table S9), though its late molt upregulation contrasts with the higher expression early in the molt in the snowshoe hare (Figure 3b).

Using qPCR, we note a tendency for higher expression of *ASIP* hair cycle isoform early in the molt of individual A, a marginal difference between early and the other stages for individual B, and no difference for individual C, though none of these patterns was significant (Figure S6, Tables S17 and S18). No significant differences in *ASIP*’s expression along the molt were detected based on RNA‐sequencing data (Figure S6).

## 4 DISCUSSION

Understanding how changes at different levels of molecular organization (e.g., gene, protein, or pathway) affect phenotype variation is critical to deciphering the evolution of adaptive phenotypes (e.g., Dalziel, Rogers, & Schulte, 2009). When possible, mechanistic studies of phenotypes should be based on natural populations so that molecular variation can be tied to the natural conditions that individuals experience (Alvarez, Schrey, & Richards, 2014) and, ultimately, to fitness (Barrett & Hoekstra, 2011). However, field‐based quantitative studies are challenging given the multiplicity of uncontrolled factors, including individual variation, that may hinder biologically meaningful patterns (Todd et al., 2016). We leveraged the ability to sample different stages of the developmental timeline of seasonal coat color change in single molting hares to control for individual variation and then combine transcriptional data from mountain and snowshoe hares to build a working model of the genic regulation of this crucial seasonal adaptation.

### 4.1 The transcriptional landscape of the molt in the context of the hair growth cycle

In keeping with the results in snowshoe hares (Ferreira et al., 2017), we found that most genes show a pattern of expression that is correlated with early (“brown”) or late (“white”) molt (Figure 3a), underlining the difficulty in discretizing the “intermediate” stage (Figures 2, 3 and S4). As in the snowshoe hare spring molt, most of the upregulated genes were detected early in the molt. This pattern of induction is expected during the proliferative stage of the hair growth cycle, anagen (Lin et al., 2004; Schlake et al., 2004), when melanogenesis occurs (Slominski & Paus, 1993). Genes upregulated in brown/early molt (cluster 1 and 2 of Figure 3a) showed enrichment of functional categories that are characteristic of anagen (Figure 3b and S10, S11, S13, and S14). For example, genes in cluster 1 and 2 were enriched in functions related with tissue organization and morphogenesis (e.g., “cytoskeleton organization,” “supramolecular fiber organization,” “tissue development”; Lin et al., 2004), gene expression and protein synthesis (e.g., “positive regulation of gene expression,” “metabolism of RNA”; Geyfman et al., 2012) cell–cell communication and adhesion (e.g., “regulation of cell communication by electrical coupling”; pathway “integrin cell surface interactions”; Botchkarev & Kishimoto, 2003; Mayer et al., 1993), and metabolism (e.g., “glycogen metabolism”; Geyfman et al., 2015; Shipman, Chase, & Montagna, 1955). An inspection of candidate genes for molt, circadian rhythm and pigment regulation, revealed that genes with known anagen‐related expression were also highly expressed early in the molt, such as hair keratins (*KRT71*; Stark, Breitkreutz, Limat, Bowden, & Fusenig, 1987; Porter et al., 2001), hair proliferation and shape‐determining genes (*EDAR* and *DKK4*; Millar, 2002; Fliniaux, Mikkola, Lefebvre, & Thesleff, 2008; Adhikari et al., 2016), genes involved in the incorporation of collagen to maintain skin integrity (*SPARC*, Table S16; collagen genes IIA1, IIIA1 and VIA2 annotated to “integrin cell surface interactions,” Table S14; Parakkal, 1969; Mayer et al., 1993; Strandjord, Madtes, Weiss, & Sage, 1999), and pigmentation genes (*EN1, SPARC, EDAR* and *MEF2C*; Table S16; Crocker & Cattanach, 1979; Slominski & Paus, 1993; Loomis, Kimmel, Tong, Michaud, & Joyner, 1998; Agarwal et al., 2011; Ditommaso et al., 2014).

Thus, the brown early molt stage largely recovers anagenic processes. However, we also detected some clear exceptions, such as the upregulation of pathways related to cell death usually characteristic of catagen (Lin et al., 2004; Stenn & Paus, 2001) and the upregulation of muscle‐related genes at the beginning of molt (Tables S10, S11, S13, and S14). Notably, muscle‐related genes were also differentially upregulated early in the spring molt of the snowshoe hare (Ferreira et al., 2017; Figure 3b), which could be related with the remodeling of tissue types that surround the hair follicle during hair growth cycle (Schmidt & Horsley, 2012).

Our white/late molt skin samples showed expression signatures consistent with hair follicles transitioning into late anagen and possibly catagen (Figures 2 and 3, Tables S12 and S15), mirroring patterns of the snowshoe hare molt (Figure 3; Ferreira et al., 2017). For instance, genes important for hair shaft differentiation and development (Table S16 *;* Lin et al., 2009), keratinization (Pinkus, Iwasaki, & Mishima, 1981), and lipid‐related functions (Tables S12 and S15) (Lin et al., 2009) were all enriched for late upregulation. Finally, catagen induction is signaled by the enrichment of retinoid related pathways (Table S15) and late molt upregulation of *STRA6* (annotated to “regulation of behavior”; Table S12), responsible for the uptake of vitamin A into the cell which seems to be important for catagen initiation (Everts, 2012; Foitzik, Spexard, Nakamura, Halsner, & Paus, 2005).

Despite the natural noise of expression studies performed in wild specimens, we were able to recover strong signatures that associate visible markers of molt progression (i.e., change in hair color) with the underlying hair cycle stages of anagen and catagen. Among this and the snowshoe hare study (Ferreira et al., 2017), we found significant overlaps among genes expressed in the molting skin (75%), among genes with stronger expression early in the molt (25% overlap), and among candidate genes for regulation of molt functions. These results suggest conservatism of the pathways and genes involved in skin processes, molt phenology, hair cycle activation (early molt) and regulation (candidate genes) in mountain and snowshoe hares, despite differences in the sampled species and molting season. The conservation of regulatory processes underlying pelage molts in these species is expected given the general conservatism of the hair growth cycle mechanism in mammals (Stenn & Paus, 2001) and the recent divergence between mountain and snowshoe hares (4 million years (Matthee et al., 2004); ~1% uncorrected pairwise sequence divergence, (Jones et al., 2018)) (Uebbing et al., 2016; Voolstra, Tautz, Farbrother, Eichinger, & Harr, 2007). The developmental framework used here should be transferable to other seasonally molting species, including other species that have evolved seasonal camouflage, thus contributing to the general understanding of the regulatory underpinnings of this adaptive phenotype across taxa (Manceau, Domingues, Linnen, Rosenblum, & Hoekstra, 2010; Rosenblum, Parent, & Brandt, 2014).

### 4.2 Candidate genes associated with circadian rhythm and pigmentation

To better understand the mechanistic regulation of brown to white autumn color change in mountain hares, we also inspected the expression of candidate genes associated with circadian rhythm regulation and pigmentation. While circadian gene expression is usually associated with late telogen and early anagen (Geyfman et al., 2015; Lin et al., 2009), in our work only one gene with “circadian rhythm” GO term annotation, *MC3R*, was found differentially expressed and upregulated toward the end of molt (Figure 3b; Table S16). *MC3R* is not a central clock gene and rather acts as a reinforcer of rhythm behavior (reviewed in Girardet, Begriche, Ptitsyn, Koza, and Butler (2014)) by regulating the expression of clock genes in response to nutrient availability and light cues (Sutton et al., 2008). Likewise, genes related to rhythmic gene expression in response to metabolic cues (Helfer et al., 2012; Oh et al., 2016; Slominski et al., 2003; Xu et al., 2009) were differentially expressed and upregulated late in molt (e.g., *APOE*, *STRA6*, and *HTR2B* in Table S12). Collectively, these genes may integrate the response to external or internal cues, such as food intake or nutritional condition, and cyclic phenotypic changes in the body, such as the molt cycle. In snowshoe hares, most of the differentially expressed circadian rhythm genes were upregulated early in the molt, and only one is known to be involved in the regulation of the circadian clock in response to nutrient intake (*GFPT1* in Figure 3b; Ferreira et al., 2017).

Given that the autumn molt of mountain hares is accompanied by growing hair changing from brown to white, we looked for expression changes of known pigmentation genes. We first focus on the pigmentation gene *ASIP* (Lu et al., 1994; Manceau, Domingues, Mallarino, & Hoekstra, 2011), which tracks the onset of melanogenesis during the spring molt of the snowshoe hare (Ferreira et al., 2017) and determines the expression of alternative winter coat colors in snowshoe and mountain hares (Giska et al., 2019; Jones et al., 2018). *ASIP’s* expression trended higher early in the molt (Figure S6), consistent with activation of melanogenesis (Slominski & Paus, 1993). However, there was no significant difference in *ASIP*’s expression among stages, both using RNA‐sequencing and targeted qPCR data (Figure S6) and, therefore, the relevance of the trend needs further investigation. The fact that *ASIP* is upregulated over a narrow window of 2–3 days during the hair growth cycle (Bultman, Michaud, & Woychik, 1992; Miller et al., 1993) may explain failure to detect the expression peak in some individuals.

Additionally, we detected early upregulation of four genes involved in pigmentation (*EN1, SPARC,* and *EDAR*) or melanocyte differentiation (*MEF2C*) (Figure 3b; Table S16). *SPARC* and *EDAR* play major roles in hair follicle and skin related processes (Adhikari et al., 2016; Fliniaux et al., 2008; Millar, 2002; Strandjord et al., 1999). *MEF2C* is involved in the differentiation of follicular melanocytes of the new hair cycle (Tobin, Slominski, Botchkarev, & Paus, 1999), and its loss results in decreased pigmentation (Agarwal et al., 2011). *EN1* is involved in establishing the boundary of expression of dorsal genes (Candille et al., 2004; Cygan, Johnson, & McMahon, 1997; Davis, Holmyard, Millen, & Joyner, 1991) and limiting the migration and proliferation of melanocytes in the ventral limb (Cygan et al., 1997). *EN1* typically shows ventral expression, and thus, the upregulation early in the molt in our dorsal samples appears surprising. It is possible that transient expression of *EN1* contributes to winter whitening, by allowing dorsal expression of the ventral isoform of *ASIP*. However, the expression of ventral *ASIP* isoform in the dorsal skin was negligible (Figure S6c; Table S17). Thus, as with snowshoe hares (Jones et al., 2018), expression of ventral *ASIP* isoform does not appear to be involved in the evolution of dorsal winter whitening in mountain hares.

There was no overlap of pigmentation candidate genes differentially expressed in mountain hares and snowshoe hares (Figure 3b). This may be due to season or species‐specific differences in expression. However, since we found a general conservation of the genic regulation of the early molt across species, we hypothesize that this difference may be related with the mechanisms of production of brown or white hairs across seasons. For example, we found upregulation of genes related with melanocyte differentiation early in autumn molt, which were not detected in spring molt (Figure 3b; Table S16; Ferreira et al., 2017), suggesting that the production of winter white coats may involve an impact on melanocyte differentiation. Whether this hypothesis can indeed be generalized from mountain to snowshoe hare must await the study of the autumn molt in the latter species and a better understanding of the genetic basis of the evolution of winter white coats in *Lepus.*

## 5 CONCLUSIONS AND SIGNIFICANCE

Coat color change is a complex adaptation to environmental seasonality that involves the regulation of circadian response, hair shedding and regrowth, and pigmentation. The joint analysis of the transcriptional landscape of the coat color molt in snowshoe and mountain hares shows that hair color in molting animals broadly delimits the underlying developmental timeline of seasonal molts. Particularly, early molt is the most transcriptionally active stage, marking the activation of circadian clock genes and inducing hair growth and pigmentation. As this inductive wave recedes, color change becomes noticeable, and in late molt a second transcription wave occurs, marking the transition from anagen to catagen. We now show that, in autumn, the upregulation of genes related with integration of nutritional cues into the circadian clock late in molt may further adjust molt rate in response to nutritional condition. This comprehensive mechanistic baseline will be a useful tool to guide functional molecular studies on the control of seasonal color change in hares and other species and to interpret quantitative genotype‐to‐phenotype studies on the genetic basis of winter coat color determination.

## CONFLICT OF INTERESTS

The authors declare no conflict of interests.

## AUTHOR CONTRIBUTIONS

JM‐F, JMG, KH, LSM, and PCA conceived the study; JM‐F, JMG, and MSF designed the work; HJ and KH organized and performed field work; CMC and LF performed laboratory work; MSF analyzed data; MSF and IG analyzed qPCR data; MSF, JM‐F, and JMG wrote the manuscript with comments from the other authors; all authors read, revised, and approved the final manuscript.

## Data Availability

Raw reads were deposited at the Sequence Read Archive (SRA) under BioProject PRJNA590529. Raw and filtered transcriptome assembly, the blast output annotations of the transcriptome to the ENSEMBL 92 rabbit (OryCun2.0) and mouse (GRCm38) references and gene and isoform level counts were deposited at Dryad (https://doi.org/10.5061/dryad.x0k6djhfh). The pipeline and custom scripts used to generate and annotate the assembly, generate bam files and do a Gene Ontology analysis with a custom annotation are available at https://github.com/evochange/coat_color_change_transcriptomics.
