# Supplementary material for: Transcriptomic regulation of seasonal coat color change in hares
Source: Ecol Evol. 2020 Jan 15;10(3):1180–92. doi: 10.1002/ece3.5956 (PMC7029059; doi:10.1002/ece3.5956)
Supplement: Supplementary file 1 [file ECE3-10-1180-s001.pdf]

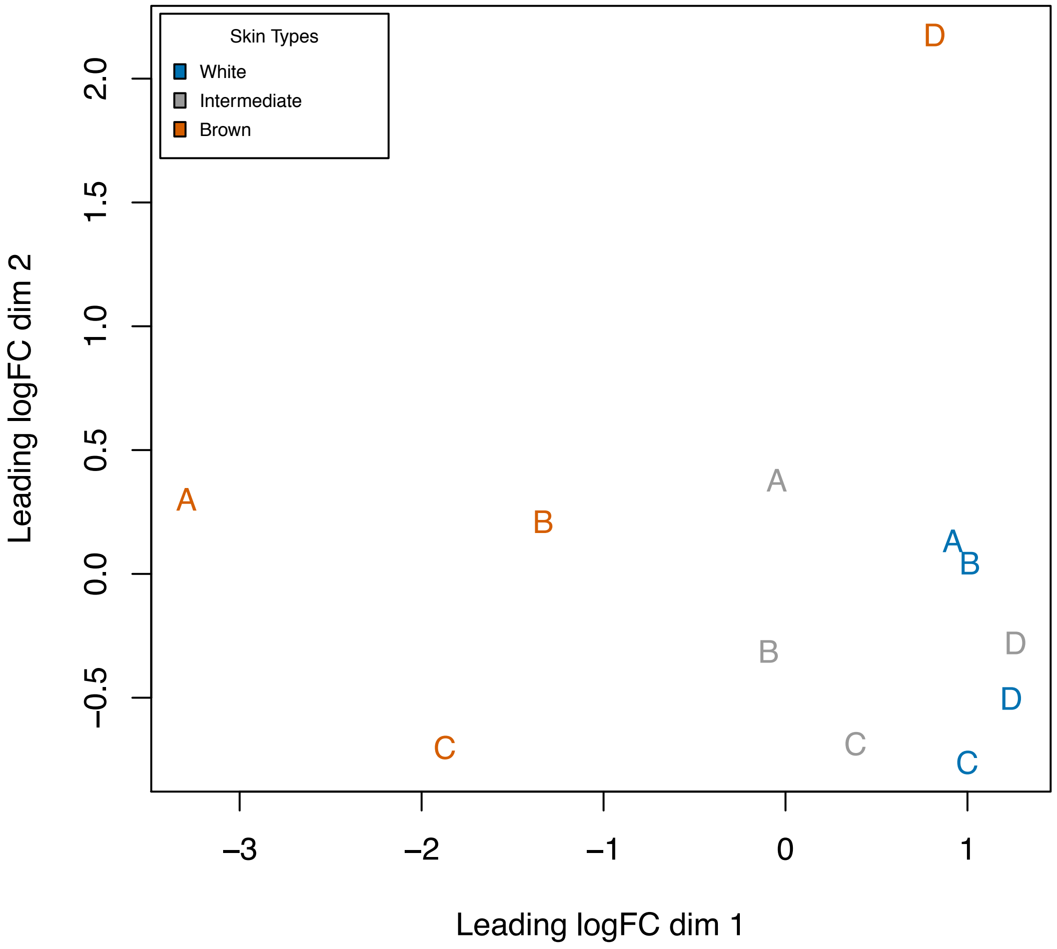


**Figure S1** - Multidimensional scaling plot with 4 individuals where distance between samples was calculated using the 500 genes with higher dispersion between each sample pair. Distance between samples is the Euclidian distance (average root-mean-square) of the 500 genes' absolute log 2 fold change between samples. Letters identify each individual sample, and colors identify each molting stage.

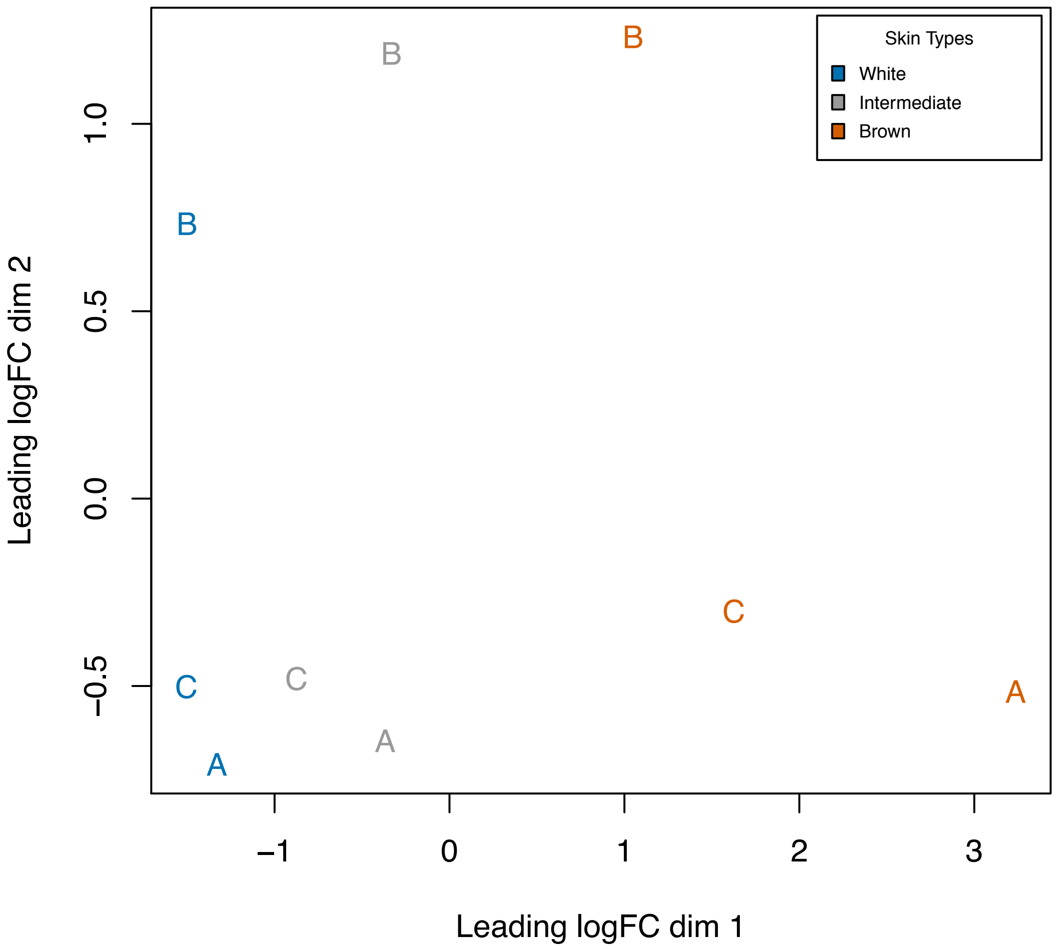


**Figure S2** - Multidimensional scaling plot with 3 individuals where distance between samples was calculated using the 500 genes with higher dispersion between each sample pair. Distance between samples is the Euclidian distance (average root-mean-square) of the 500 genes' absolute log 2 fold change between samples. Letters identify each individual sample, and colors identify each molting stage.

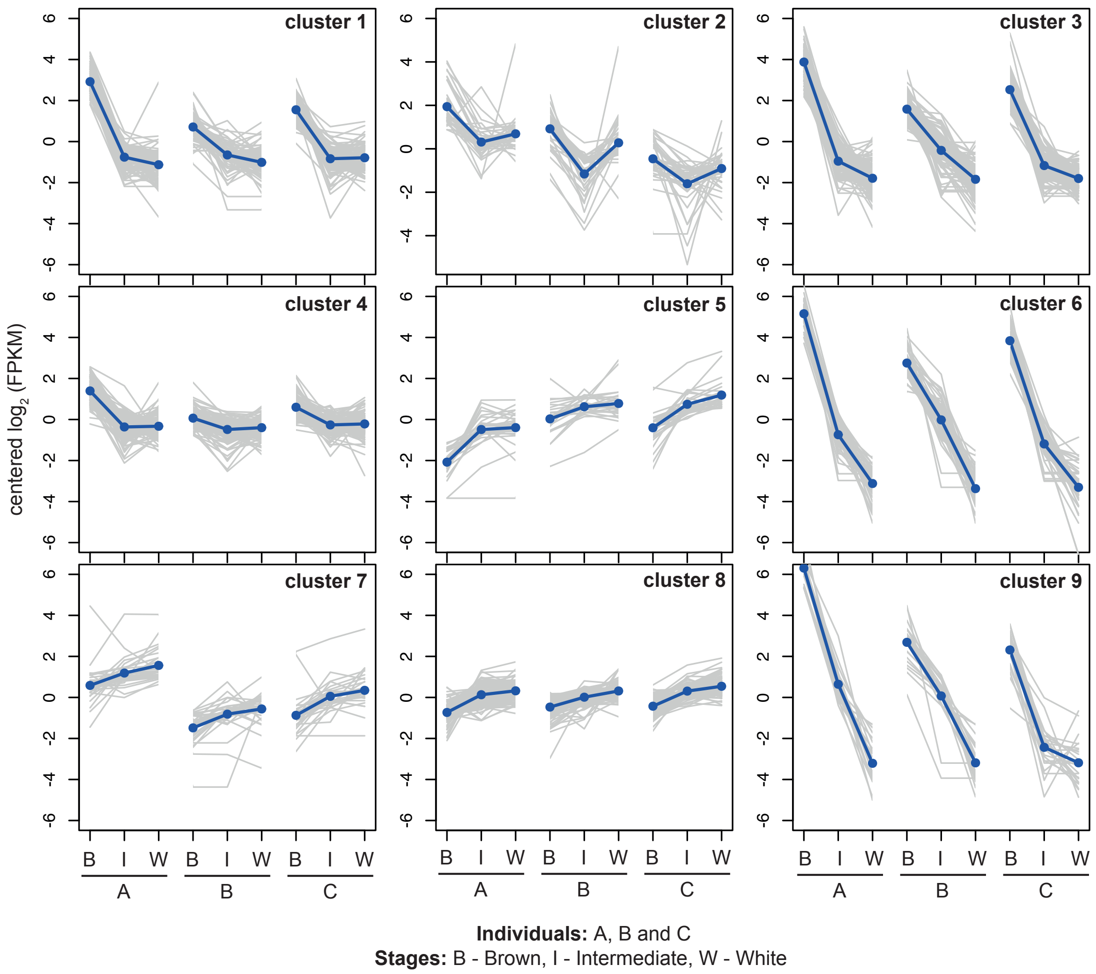


**Figure S3** - Clusters are resultant from PAM clustering of individual gene expression levels for k=9. The average expression level for the group of genes in each cluster is displayed in black. Letters A, B and C represent mountain hare individuals and letters B, I and W represent the "brown", "intermediate" and "white" molting stages, respectively.

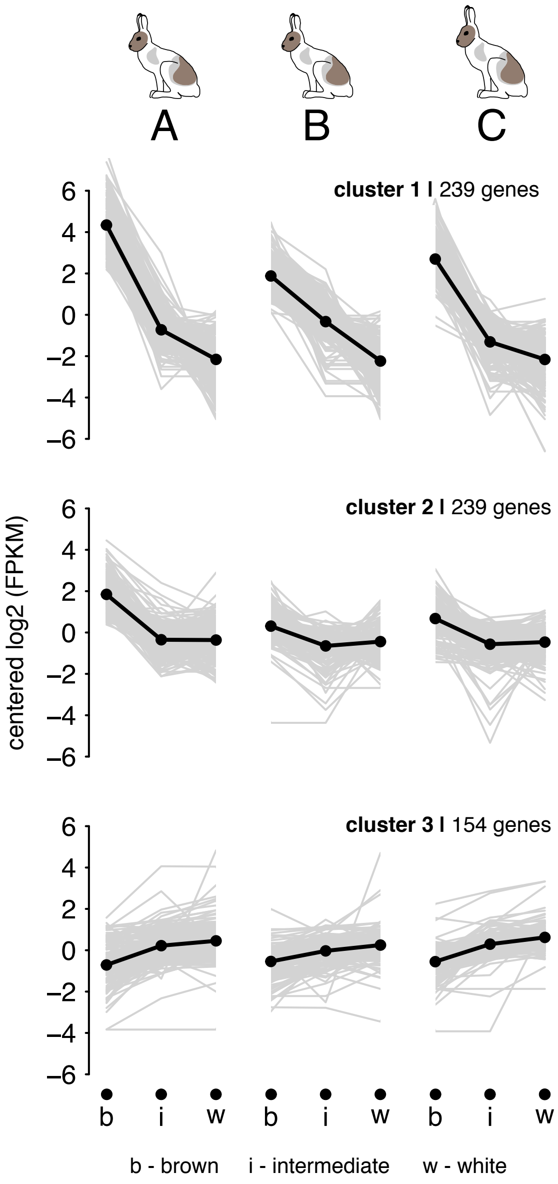


**Figure S4** - Clusters are resultant from PAM clustering of individual gene expression levels for k=3. The average expression level for the group of genes in each cluster is displayed in black. Letters A, B and C represent mountain hare individuals and letters B, I and W represent the "brown", "intermediate" and "white" molting stages, respectively.

| | Gene Ontology Biological Process term | Mountain hare cluster | Snowshoe hare cluster |
|---|---|---|---|
| **GO:0030029** | actin filament-based process | 1,2 | 2 |
| **GO:0060402** | calcium ion transport into cytosol | 1 | 2 |
| **GO:0007010** | cytoskeleton organization | 1 | 2 |
| **GO:0007507** | heart development | 1 | 2 |
| **GO:0006811** | ion transport | 1 | 2 |
| **GO:0032501** | multicellular organismal process | 1 | 1 |
| **GO:0055001** | muscle cell development | 1 | 2 |
| **GO:0042692** | muscle cell differentiation | 1 | 2 |
| **GO:0007517** | muscle organ development | 1 | 2 |
| **GO:0048644** | muscle organ morphogenesis | 1 | 2 |
| **GO:0061061** | muscle structure development | 1,2 | 2 |
| **GO:0003012** | muscle system process | 1 | 2 |
| **GO:0060537** | muscle tissue development | 1 | 2 |
| **GO:0060415** | muscle tissue morphogenesis | 1 | 2 |
| **GO:0051283** | negative regulation of sequestering of calcium ion | 1 | 2 |
| **GO:0043462** | regulation of ATPase activity | 1 | 2 |
| **GO:0051282** | regulation of sequestering of calcium ion | 1 | 2 |
| **GO:0070296** | sarcoplasmic reticulum calcium ion transport | 1 | 2 |
| **GO:0003008** | system process | 1 | 2 |
| | **Reactome pathways** | | |
| **R-MMU-390522** | Striated Muscle Contraction | 1 | 2 |
| **R-MMU-397014** | Muscle contraction | 1,2 | 2 |
| **R-MMU-70221** | Glycogen breakdown (glycogenolysis) | 2 | 2 |

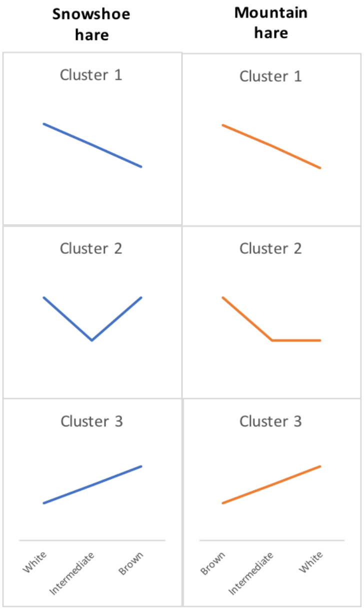


**Figure S5** - Functional overlap between mountain hare Fall molt and snowshoe hare Spring molt dataset. Overlapping Gene Ontology Biological Process term and REACTOME pathway and the clusters where they are enriched in both species datasets.

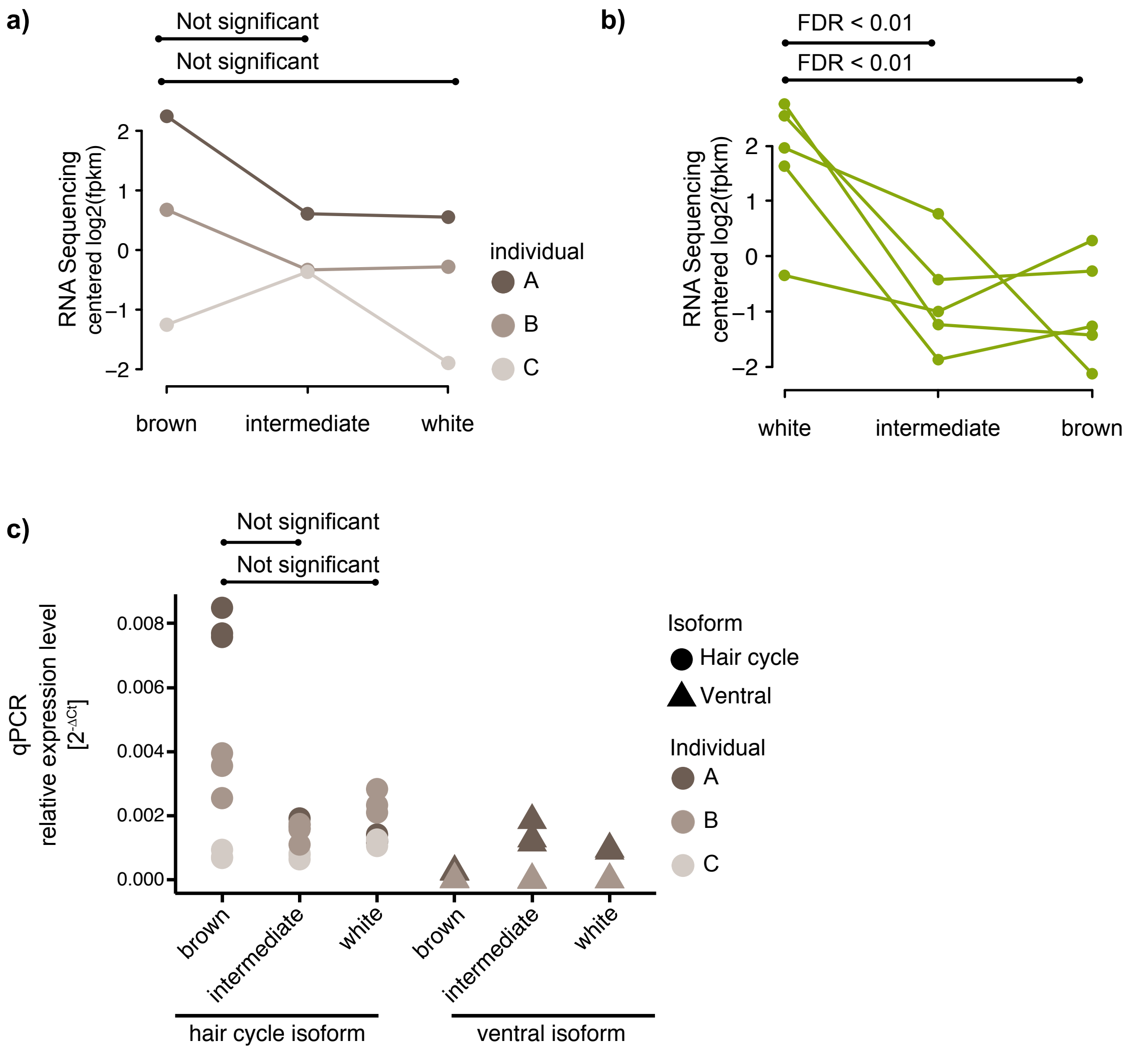


**Figure S6** – The expression of candidate agouti signaling peptide (*ASIP*) gene across the autumn molt of (A, C) mountain hares (this work) and (B) spring molt of snowshoe hares (recalculated from Ferreira et al. 2017). For both species (A, B), expression levels are shown in mean centered log2 FPKM for all individuals in each dataset, derived from RNA-sequencing. For mountain hares, (C) relative expression levels estimated from qPCR ($2^{-\Delta Ct}$) for the *ASIP* hair cycle and ventral isoform are shown for individuals and tissue types, using *ACTB* as a reference gene.
