# Supplementary material for: Transcriptomic regulation of seasonal coat color change in hares
Source: Ecol Evol. 2020 Jan 15;10(3):1180–92. doi: 10.1002/ece3.5956 (PMC7029059; doi:10.1002/ece3.5956)
Supplement: Supplementary file 2 [file ECE3-10-1180-s002.pdf]

Table S1 - Sampling details for the individuals used in this study.

Individual	Database ID	Sampling location	Sex	% White
A	3112	Falein, Filisur, Switzerland	F	10%
D	3113	Falein, Filisur, Switzerland	F	5%
B	3114	Trimmis, Stams, Switzerland	M	25%
C	3116	Aienfeld, Guscha, Switzerland	F	40%

Table S2 - Sequences, sequencing efficiencies and expected PCR product length of the primers used for qPCR expression analysis of the ventral and hair cycle *ASIP* isoforms from Jones et al. 2018, Science, and reference genes *ACTB* (from Fabiana Neves, CIBIO-InBIO, University of Porto) and *SDHA* (from Giska et al. 2019, PNAS).

Product	Primer	Sequence (5'-3')	Efficiency [%]	PCR product [bp]
ASIP hair cycle isoform	agouti_hair_F3	GGACAGAAAAGACGCTCTGC	96% (R^2 = 0.9968)	180
	agouti_trscpt_R2	TGGTCATCAGTGGGTTTCTCC		
ASIP ventral specific isoform	agouti_vent_F4	TGGAAGAGGGAGTCATTAGCTG	107% (R2 = 0.9934)	163
	agouti_trscpt_R2	TGGTCATCAGTGGGTTTCTCC		
ACTB	LTM_actb_F	GAAGATCTGGCACCACACCTTC	118% (R^2 = 0.924)*	162
	LTM_actb_R	CCTGGATGGCCACGTACATG		
SDHA	LTM_sdha_F	CCCTTGTAGTTGGTAGGAATGC	114% (R^2 = 0.971)	181
	LTM_sdha_R	CCCGACAAGGATCACGTCTA		

* efficiency estimated simultaneously with analysis from Giska et al. 2019, PNAS.

Table S3 - Thermal conditions for quantitative PCR used to amplify *ASIP* isoforms and reference genes.

	Temperature (°C)	Time (s)
	95	30
40 cycles	95	5
	62	30
melt curve analysis	65 to 95, in 0.5°C increments	

Table S4 - Number of reads for each individual skin sample, before and after filtering for quality.

Samples	Number of raw reads	Number of filtered reads
A brown skin	11 225 550	10 064 999
A intermedite skin	11 534 533	10 395 094
A white skin	11 622 112	10 349 562
B brown skin	11 361 420	10 171 463
B intermediate skin	11 682 515	10 423 361
B white skin	12 255 152	10 946 710
C brown skin	12 056 015	10 670 922
C intermediate skin	11 747 125	10 526 823
C white skin	11 799 652	10 518 387
D brown skin	11 328 248	10 180 415
D intermediate skin	11 418 692	10 229 305
D white skin	10 974 557	9 916 123
**Total**	139 005 571	124 393 164

Table S5 - Transrate metrics for the raw transcriptome produced with Trinity.

Transrate Metrics	Raw Transcriptome Value
No. Contigs	233182
Size Smallest Contig	201
Size Largest Contig	20708
No. Assembled bases	183897734
Contig mean length	788.64464
No. contigs shorter than 200bp	0
No. contigs longer than 1k bp	47338
No. contigs longer than 10k	158
No. contigs with Open Reading Frame	50920
% of contig length covered by ORF	58.63364
N90	271
N70	698
N50	1714
N30	2994
N10	5251
% GC bases	0.51768
GC skew	0.01606
AT skew	0.01445
Count o CpG sites relative to expected	1.55914
No. of N bases	0
Proportion of N Bases	0
Linguistic Complexity	0.13407
No. read paires provided	124393164
No. of read pairs mapped	106605225
Proportion of mapped reads	0.857
No. Of read pairs mapped indicative of good assembly	77318085
Proportion of read pairs mapped indicative of good assembly	0.62156
No. Of read pairs mapped indicative of bad assembly	29287140
No. Of potential links between contigs	43755
No. Bases not covered by any reads	21364227
Proportion of bases not covered by any reads	0.11617
No. of contigs that contain bases with no read coverage	89989
Prop. of contigs that contain bases with no read coverage	0.38592
No. of contigs that contain bases with mean-read coverage <1	18509
Prop. of contigs that contain bases with mean-read coverage <1	0.07938
No. of contigs that contain bases with mean-read coverage <10	181553
Prop. of contigs that contain bases with mean-read coverage <10	0.77859
the number of contigs that have >=50% estimated chance of being segmented	17258
the proportion of contigs that have >=50% estimated chance of being segmented	0.07401
Assembly score	0.22641
Optimal assembly score	0.30087
Cutoff	0.15904
weighted	0.83561
Number of good contigs	202135
Proportion of good contigs	0.87

Table S6 - Filtering steps applied to the transcriptome, and the number of Trinity genes retained in each step.

Filtering steps	Trinity genes
Raw transcriptome	173848
Transrate good transcriptome	159769
Annotated transcriptome	36101
Expression filter	17270
