# Supplementary material for: Transcriptomic regulation of seasonal coat color change in hares
Source: Ecol Evol. 2020 Jan 15;10(3):1180–92. doi: 10.1002/ece3.5956 (PMC7029059; doi:10.1002/ece3.5956)
Supplement: Supplementary file 4 [file ECE3-10-1180-s004.pdf]

Table S16 - Information about the genes annotated to the specific Gene Ontology Biological Process categories in the first and second column. For each gene we show the respective Trinity "gene" code, log fold change and significance value (FDR) for each pairwise comparison (BSWS – Brown vs White; ISWS – Intermediate vs White; BSIS – Brown vs Intermediate). Also, for each gene the annotation to the ENSEMBL database (ENSEMBL gene code) is given.

**Pigmentation (GO:0043743)**		**Annotation**	**Gene name**	**Trinity code**	**logFC ISWS**	**FDR**	**logFC BSIS**	**FDR**	**logFC BSWS**	**FDR**	**Cluster**
GO:0043473	pigmentation	ENSOCUG00000011901	EN1	TRINITY_DN79469_c8_g5	-1.61	1.00	-0.80	0.93	-2.41	0.02	2
GO:0043473	pigmentation	ENSOCUG00000012120	SPARC	TRINITY_DN81045_c0_g3	0.58	1.00	-1.50	0.02	-0.92	0.38	2
GO:0043473	pigmentation	ENSOCUG00000025015	EDAR	TRINITY_DN85082_c0_g1	-0.52	1.00	-0.59	0.44	-1.11	0.00	2
GO:0030318	melanocyte differentiation	ENSOCUG00000029354	MEF2C	TRINITY_DN79548_c0_g6	-0.32	1.00	-1.17	0.11	-1.49	0.01	2
**Molting cycle (GO:0042303)**											
GO:0031069	hair follicle morphogenesis	ENSMUSG00000038415	Foxq1	TRINITY_DN71926_c0_g2	-0.16	1.00	1.10	0.00	0.94	0.01	3
				TRINITY_DN78042_c1_g1	-0.03	1.00	0.74	0.04	0.71	0.06	3
GO:0061170	negative regulation of hair follicle placode formation	ENSOCUG00000001298	DKK4	TRINITY_DN75230_c0_g1	-1.47	0.13	-0.31	1.00	-1.78	0.00	2
GO:0031069	hair follicle morphogenesis	ENSOCUG00000029234	KRT71	TRINITY_DN78709_c0_g2	0.39	1.00	-1.13	0.03	-0.74	0.35	2
GO:0042633	hair cycle	ENSOCUG00000011781	KRT16	TRINITY_DN79269_c2_g1	0.20	1.00	0.34	0.49	0.54	0.03	3
				TRINITY_DN85157_c7_g3	0.23	1.00	0.47	0.11	0.70	0.00	3
GO:0001942	hair follicle development	ENSOCUG00000025015	EDAR	TRINITY_DN85082_c0_g1	-0.52	1.00	-0.59	0.44	-1.11	0.00	2
**Circadian rhythm (GO:0007623)**											
GO:0032922	circadian regulation of gene expression	ENSOCUG00000010002	MC3R	TRINITY_DN74471_c1_g2	0.05	1.00	2.43	0.01	2.48	0.01	3
GO:0045475	locomotor rhythm										

Table S17 - Quantitative PCR results for the amplifications of *ASIP* (*Agouti*) hair cycle and ventral isoforms. The raw qPCR results, presented as the threshold cycle (Ct), were used to calculate expression of each *ASIP* isoform, normalized by the expression of the *ACTB* or *SDHA* genes according to the formula $2^{(-\Delta Ct)}$, where $\Delta Ct = Ct^{Agouti} - Ct^{reference\ gene}$. We performed calculations by subtracting the mean Ct of the reference gene from each technical replicate Ct of the *ASIP* gene, to present the relative expression as normalized individual data points. Results of in the main text and in Fig. S6 are reported based on *ACTB* gene.

Hair cycle isoform

											ACTB		SDHA	
Molt	Individual	agouti CT	Mean agouti	SD agouti	ACTB Ct	Mean ACTB	SD ACTB	SDHA Ct	Mean SDHA	SD SDHA	Δ Ct	2^(-ΔCt)	Δ Ct	2^(-ΔCt)
	A	23.19	23.15	0.09	16.04	16.17	0.16	22.34	22.31	0.02	7.022854	0.007690	0.879600	0.543518
	A	23.05			16.35			22.32			6.880146	0.008489	0.736892	0.600031
	A	23.21			16.12			22.29			7.043735	0.007579	0.900481	0.535708
	B	25.88	25.51	0.33	17.31	17.27	0.24	24.04	23.95	0.07	8.613586	0.002553	1.927058	0.262965
brown	B	25.25			17.01			23.91			7.983586	0.003951	1.297058	0.406955
	B	25.40			17.48			23.91			8.133586	0.003561	1.447058	0.366769
	C	26.77	26.66	0.25	16.37	16.32	0.05	22.93	23.06	0.11	10.453558	0.000713	3.716468	0.076073
	C	26.37			16.29			23.12			10.053735	0.000941	3.316645	0.100367
	C	26.83			16.30			23.12			10.507645	0.000687	3.770555	0.073274
	A	25.40	25.54	0.13	16.51	16.38	0.11	22.35	22.40	0.06	9.025056	0.001919	3.005089	0.124560
	A	25.56			16.31			22.38			9.183401	0.001720	3.163433	0.111612
	A	25.66			16.31			22.46			9.279063	0.001610	3.259096	0.104451
	B	25.96	25.57	0.35	16.35	16.14	0.23	22.80	22.77	0.20	9.822480	0.001104	3.193398	0.109318
intermediate	B	25.30			16.18			22.56			9.162203	0.001745	2.533121	0.172765
	B	25.44			15.89			22.95			9.303062	0.001583	2.673980	0.156694
	C	25.40	25.54	0.19	15.25	15.15	0.20	21.60	21.60	0.00	10.244767	0.000824	3.801487	0.071720
	C	25.75			14.93			21.60			10.598774	0.000645	4.155494	0.056114
	C	25.48			15.29			21.60			10.321330	0.000782	3.878050	0.068013
	A	25.74	25.90	0.14	16.34	16.28	0.10	22.35	22.40	0.06	9.461443	0.001418	3.345327	0.098391
	A	26.01			16.34			22.38			9.726091	0.001181	3.609976	0.081901
	A	25.94			16.16			22.46			9.663872	0.001233	3.547756	0.085510
	B	25.28	25.09	0.22	16.36	16.39	0.03	22.59	22.68	0.08	8.892688	0.002104	2.598440	0.165117
white	B	24.85			16.41			22.76			8.462688	0.002835	2.168440	0.222451
	B	25.13			16.39			22.69			8.742688	0.002334	2.448440	0.183209
	C	26.10	25.94	0.14	16.24	16.22	0.03	22.72	22.98	0.24	9.882679	0.001059	3.114218	0.115485
	C	25.85			16.18			23.05			9.631785	0.001261	2.863324	0.137421
	C	25.86			16.22			23.19			9.645482	0.001249	2.877021	0.136123

Ventral specific isoform

											ACTB		SDHA	
Molt	Individual	agouti CT	Mean agouti	SD agouti	ACTB Ct	Mean ACTB	SD ACTB	SDHA Ct	Mean SDHA	SD SDHA	Δ Ct	2^(-ΔCt)	Δ Ct	2^(-ΔCt)
	A	28.04	28.10	0.09	16.04	16.17	0.16	22.34	22.31	0.02	11.871156	0.000267	5.727902	0.018868
	A	28.20			16.35			22.32			12.025729	0.000240	5.882475	0.016951
	A	28.06			16.12			22.29			11.884233	0.000265	5.740979	0.018698
	B	32.42	32.84	0.64	17.49	17.40	0.08	24.27	24.08	0.21	15.020035	0.000030	8.337993	0.003090
brown	B	33.58			17.33			24.12			16.181657	0.000013	9.499616	0.001381
	B	32.53			17.38			23.85			15.133724	0.000028	8.451683	0.002856
	C	33.55	33.79	0.63	16.37	16.32	0.05	22.93	23.06	0.11	17.230009	0.000007	10.492918	0.000694
	C	33.32			16.29			23.12			17.000802	0.000008	10.263711	0.000813
	C	34.51			16.30			23.12			18.186256	0.000003	11.449166	0.000358
	A	26.14	25.86	0.37	16.51	16.38	0.11	22.35	22.40	0.06	9.765915	0.001149	3.745947	0.074535
	A	25.98			16.31			22.38			9.607591	0.001282	3.587624	0.083180
	A	25.44			16.31			22.46			9.066422	0.001865	3.046455	0.121039
	B	35.37	35.00	0.34	16.61	16.57	0.04	23.23	23.25	0.02	18.804817	0.000002	12.123926	0.000224
intermediate	B	34.92			16.58			23.26			18.346819	0.000003	11.665928	0.000308
	B	34.70			16.52			23.27			18.133162	0.000003	11.452271	0.000357
	C	32.00	32.44	0.46	15.25	15.15	0.20	21.60	21.60	0.00	16.850159	0.000008	10.406879	0.000737
	C	32.93			14.93			21.60			17.775108	0.000004	11.331828	0.000388
	C	32.39			15.29			21.60			17.239210	0.000006	10.795929	0.000562
	A	26.31	26.33	0.05	16.34	16.28	0.10	22.35	22.40	0.06	10.027579	0.000958	3.911463	0.066456
	A	26.39			16.34			22.38			10.111977	0.000904	3.995861	0.062680
	A	26.29			16.16			22.46			10.011873	0.000969	3.895757	0.067183
	B	32.37	32.36	0.24	16.63	16.58	0.04	23.09	23.14	0.08	15.783020	0.000018	9.225451	0.001671
white	B	32.59			16.57			23.24			16.004449	0.000015	9.446881	0.001433
	B	32.12			16.56			23.09			15.532762	0.000021	8.975194	0.001987
	C	31.85	32.42	0.56	16.24	16.22	0.03	22.72	22.98	0.24	15.635669	0.000020	8.867208	0.002141
	C	32.45			16.18			23.05			16.239112	0.000013	9.470651	0.001409
	C	32.97			16.22			23.19			16.752882	0.000009	9.984421	0.000987

Table S18 - Linear regression test results modeling the contribution of "Individual" and "Color" to the expression levels of Agouti, using *ACTB* as a reference gene. "Brown" and "Individual A" are used as reference levels. Linear regression was performed with pcr_lm() from the R package *pcr*.

**Term**	**Estimate**	**P-value**	**Lower CI**	**Upper CI**
Individual B	0.20	0.78	-1.73	2.13
Individual C	1.56	0.09	-0.37	3.49
White	0.83	0.30	-1.10	2.76
Intermediate	1.14	0.18	-0.79	3.07
